# Cross-priming of cyclin B1, MUC-1 and survivin-specific CD8^+ ^T cells by dendritic cells loaded with killed allogeneic breast cancer cells

**DOI:** 10.1186/bcr1621

**Published:** 2006-11-27

**Authors:** Hiroaki Saito, Peter Dubsky, Carole Dantin, Olivera J Finn, Jacques Banchereau, A Karolina Palucka

**Affiliations:** 1 Baylor Institute for Immunology Research, Live Oak, Dallas, Texas 75024, USA; 2 Department of Immunology, University of Pittsburgh School of Medicine, E1040 Biomedical Sciences Tower, Pittsburgh, Philadelphia 15261, USA

## Abstract

**Introduction:**

The ability of dendritic cells (DCs) to take up whole tumor cells and process their antigens for presentation to T cells ('cross-priming') is an important mechanism for induction of tumor specific immunity.

**Methods:**

*In vitro *generated DCs were loaded with killed allogeneic breast cancer cells and offered to autologous naïve CD8^+ ^T cells in 2-week and/or 3-week cultures. CD8^+ ^T cell differentiation was measured by their capacity to secrete effector cytokines (interferon-γ) and kill breast cancer cells. Specificity was measured using peptides derived from defined breast cancer antigens.

**Results:**

We found that DCs loaded with killed breast cancer cells can prime naïve CD8^+ ^T cells to differentiate into effector cytotoxic T lymphocytes (CTLs). Importantly, these CTLs primed by DCs loaded with killed HLA-A*0201^- ^breast cancer cells can kill HLA-A*0201^+ ^breast cancer cells. Among the tumor specific CTLs, we found that CTLs specific for HLA-A2 restricted peptides derived from three well known shared breast tumor antigens, namely cyclin B1, MUC-1 and survivin.

**Conclusion:**

This ability of DCs loaded with killed allogeneic breast cancer cells to elicit multiantigen specific immunity supports their use as vaccines in patients with breast cancer.

## Introduction

Despite declining mortality rates, breast cancer ranks second among cancer related deaths in women. In the USA alone, it is estimated that more than 200,000 new cases of breast cancer will be diagnosed yearly, with that about 40,000 patients will die from breast cancer [1]. Therefore, there is a need to develop novel therapeutic approaches to improve survival rates among patients with breast cancer.

Evidence is accumulating that naturally occurring immunity is present in patients with breast cancer against tumor associated antigens such as HER-2/neu [2,3] and cdr2 [4], as well as the antigens investigated here, namely MUC-1 [5,6], cyclin B1 [7] and survivin [8]. Several clinical studies have now demonstrated that immunity against tumor antigens can also be boosted or elicited in cancer patients by vaccination, most recently through the use of tumor antigen loaded dendritic cells (DCs) [9-13]. For example, in a recently reported trial [14], two out of ten patients with breast/ovarian cancer vaccinated with HER-2/neu or MUC-1 peptide-pulsed monocyte-derived DCs generated cytotoxic T lymphocytes (CTLs) that were able to kill HLA-A*0201 cell lines expressing these antigens. The optimal source of tumor antigens for loading of *ex vivo *generated DCs is yet to be determined [15]. In particular, strategies that allow tumor antigen presentation across HLA haplotypes are needed, and several have been undergoing investigation. These include loading DCs with recombinant proteins, killed tumor cells [4,16-18], tumor RNA [19,20] and viral vectors that encode tumor antigens [21], and fusing DCs with tumor cells [22]. In prostate cancer [17] and in melanoma [18], we previously showed that DCs loaded with killed allogeneic tumor cells cross-prime naïve CD8^+ ^T cells to differentiate into CTLs specific for shared tumor antigens.

In breast cancer, we previously demonstrated that DCs loaded with killed breast cancer cells can induce CTLs that can kill those breast cancer cells [23]. However, we were unable to determine the antigen specificity of these CTLs. In an extension of that work, here we demonstrate that DCs loaded with killed allogeneic breast cancer cells can prime naïve CD8^+ ^T cells to differentiate into tumor antigen specific CTLs by confirming their specificity for three known breast cancer antigens: cyclin B1, MUC-1, and survivin.

## Materials and methods

### Media and reagents

Complete RPMI-1640 was supplemented with 10% heat-inactivated fetal calf serum (Hyclone, Logan, UT, USA) for DCs and 10% human serum AB (Gem cell) for T cell cultures. Cytokines used included granulocyte-macrophage colony-stimulating factor (100 ng/ml; Immunex, Amgen Thousand Oaks, CA, USA), IL-4 (25 ng/ml; R&D Systems, Minneapolis, MN, USA), soluble CD40 ligand (200 ng/ml; R&D Systems), IL-2 (10 IU/ml; R&D Systems), and IL-7 (10 IU/ml; R&D Systems). Betulinic acid (Sigma-Aldrich, St. Louis, MO, USA) was used at 10 mg/ml. Cyclin B1 peptide CB9 (sequence AKYLMELTM) was synthesized at the University of Pittsburgh Cancer Institute Peptide Synthesis facility. MUC-1 peptides D6 (sequence LLLTVLTVV) and PSA1 (sequence FLTPKKLQCV) were purchased from Bio-Synthesis (TX, Lewisville, USA). The surviving peptide library was synthesized at the Rockefeller University.

### Cell lines

Breast cancer cell lines T47D, Hs578T, MCF-7, natural killer cell target K562 and HLA-A*0201^+ ^T2 cells, which are targets for CTLs, were obtained from the American Type Culture Collection (Manassas, VA, USA). The Me275 melanoma cell line was a kind gift from Drs J-C Cerottini and D Rimoldi. All cell lines were maintained in complete RPMI1640 (Gibco, Invitrogen, Carlsbad, California, USA). For loading of DCs, T47D and Hs578T cells were killed by incubation with betulinic acid (Sigma-Aldrich), as described previously [18]. MCF-7 cells were killed by gamma irradiation (80 Gy) and 24 hours of exposure to tumor necrosis factor-α (100 ng/ml; R&D Systems).

### Generation of cytotoxic T lymphocytes

Naïve HLA-A*0201^+ ^CD8^+ ^T cells (autologous to the DCs) were depleted of other cells using anti-CD4, anti-CD14, anti-CD16, anti-CD19, anti-CD56, and anti-glycophorin A microbeads (Miltenyi, Auburn, CA, USA) and sorted based on CD8^+^CD45RA^+^CCR7^+^CD45RO^- ^phenotype. DCs were generated from the adherent fraction of peripheral blood mononuclear cells by culturing for 6 days in complete RPMI1640 supplemented with granulocyte-macrophage colony-stimulating factor and IL-4, loaded with killed tumor cells, sorted, and used as stimulators of autologous purified naïve CD8^+ ^T cells at a 1:10 ratio. Soluble CD40 ligand was added to induce DC maturation, and IL-7 (10 IU/ml in weeks 1, 2, and 3) and IL-2 (10 IU/ml in weeks 2 and 3) for T cell expansion. T cells were restimulated weekly with tumor cell loaded DCs.

### ^51^Cr cytotoxicity assay

For use as targets, T2 cells were pulsed overnight with 10 μg/ml of various peptides, labeled with ^51^Cr (NEN Life Science Products, Boston, Massachusetts, USA) and co-cultured with CTLs for 4 hours. Specific lysis was calculated using the following formula (where cpm is the counts/minute): % release = 100 × (cpm experiment – cpm spontaneous release)/(cpm maximum release – cpm spontaneous release). For the mAb blocking assays, anti-HLA-A, HLA-B and HLA-C antibody (W6/32; DAKO, Carpenteria, CA, USA; 5 μg/ml) or an irrelevant (control) antibody was added to the target cells 30 minutes before the addition of CTLs and left throughout the culture period.

### Tumor inhibition assay

Target tumor cell lines were suspended at a concentration of 5 × 10^4^/ml with RPMI1640 medium containing 10% AB serum and the CTLs at 10^6^/ml. Targets cells and CTLs were co-incubated in 96-U bottom plates (Costar, Corning, Sigma-Aldrich) for 24 hours. The numbers of live and dead tumor cells were determined using trypan blue exclusion.

### Intracellular cytokine staining

Primed and boosted T cells were cultured for 6 hours with DCs loaded with 15-mer peptides representing survivin peptide library. At 2 hours of stimulation, Golgi-stop™ (BD Pharmingen, San Diego, CA, USA) was added to the culture. At 6 hours of stimulation, cells were harvested and first stained with CD3-PerCP and CD8-APC mAbs, fixed and permeabilized with BD Cytofix/Cytoperm™ solution (BD), and then stained with anti-IFN-γ PE (BD Pharmingen).

### T cell restimulation assay

CD8^+ ^T cells that had undergone two stimulations with DCs loaded with killed breast cancer cells were co-incubated with autologous DCs pulsed with the survivin peptide library at a 10:1 ratio. The T cells were analyzed after an additional 5 to 7 days of culture by intracellular cytokine staining, as described above.

### Statistical analysis

Nonparametric Kruskal-Wallis analysis of variance was used as indicated to assess the killing of three different target cells. *P *< 0.05 was considered statistically significant.

## Results

### Development of breast cancer specific CD8^+ ^T cells through cross-priming

**Figure 1:**
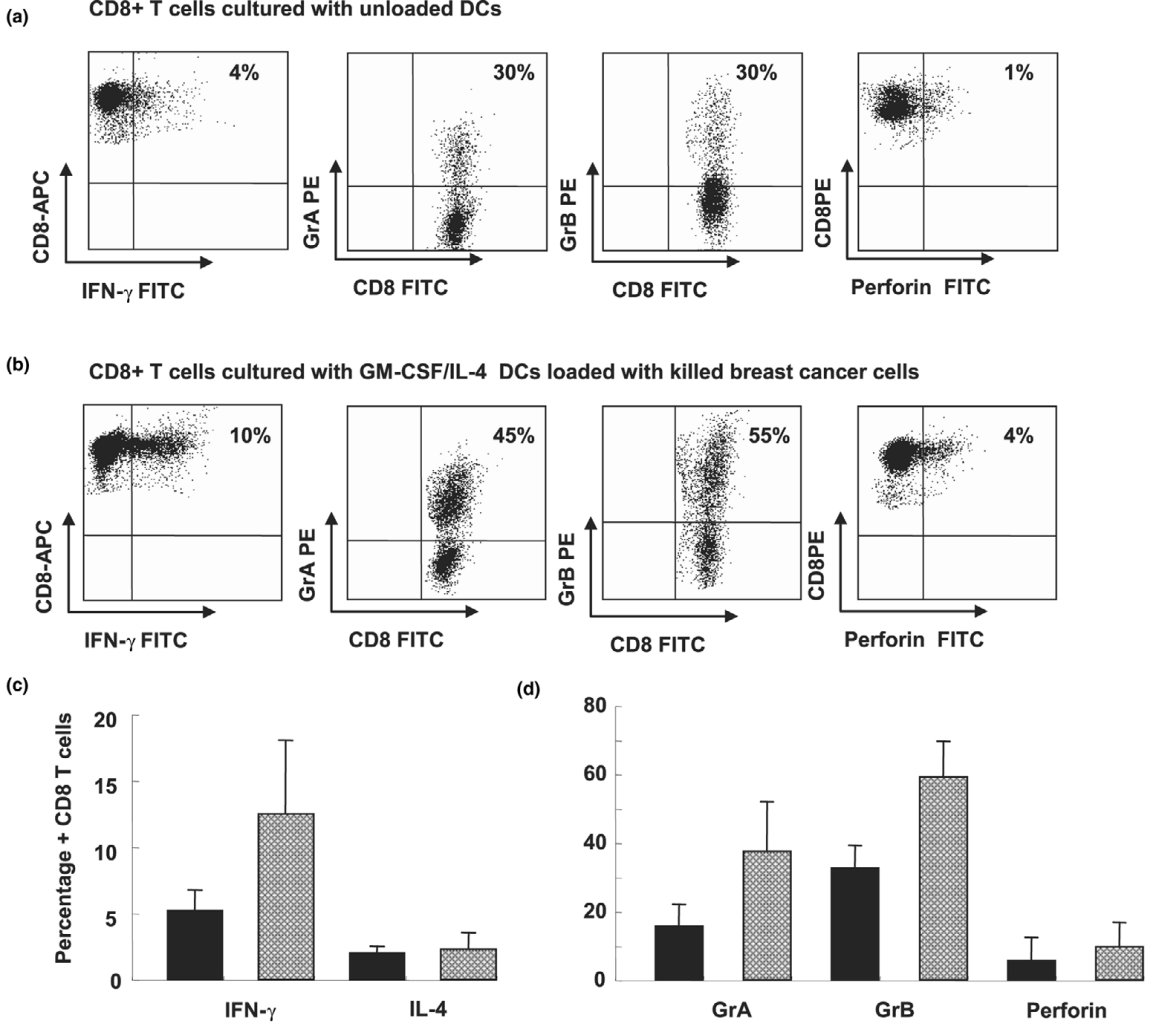
DCs loaded with killed breast cancer cells induce differentiation of naïve CD8^+ ^T cells into effector cells. Sorted naïve CD8^+ ^T were cultured for 3 weeks with autologous DCs either **(a) **not loaded or **(b) **loaded with killed breast cancer cells. The cultures were restimulated twice. Intracytoplasmic staining and flow cytometry are given for the indicated effector molecules. Shown is one representative experiment from three with T cells from three different healthy donors. Percentage of CD8^+ ^T cells expressing **(c) **indicated cytokines or **(d) **effector molecules in cultures with unloaded (black bars) and loaded (gray bars) DCs. DC, dendritic cell; GM-CSF, granulocyte-macrophage colony-stimulating factor; Gr, granzyme; IFN, interferon; IL, interleukin.

We used HLA-A*0201^+ ^monocyte-derived DCs loaded with killed allogeneic breast cancer cells to prime *in vitro *autologous naïve CD45RA^+^CCR7^+^CD45RO^-^CD8^+ ^T cells. The priming and weekly restimulations were performed with the addition of soluble CD40 ligand, and the T cells were maintained in the presence of 10 U/ml of cytokines IL-7 and IL-2. As shown in Figure 1, after 3 weeks of culture naïve CD8^+ ^T cells differentiated into effector cells, as measured by expression of IFN-γ, granzymes A and B, and perforin (Figure 1a). This differentiation was observed in cultures stimulated with control DCs as well as antigen loaded DCs, but the antigen loaded DCs stimulated greater expansion of effector CD8^+ ^T cells expressing granzymes A and B (Figure 1b to 1d). A possible explanation for the antigen-independent differentiation seen in several independent experiments using T cells from three different donors could come from the addition of T cell cytokines such as IL-7 and IL-2 to support CD8^+ ^T cell priming.

**Figure 2:**
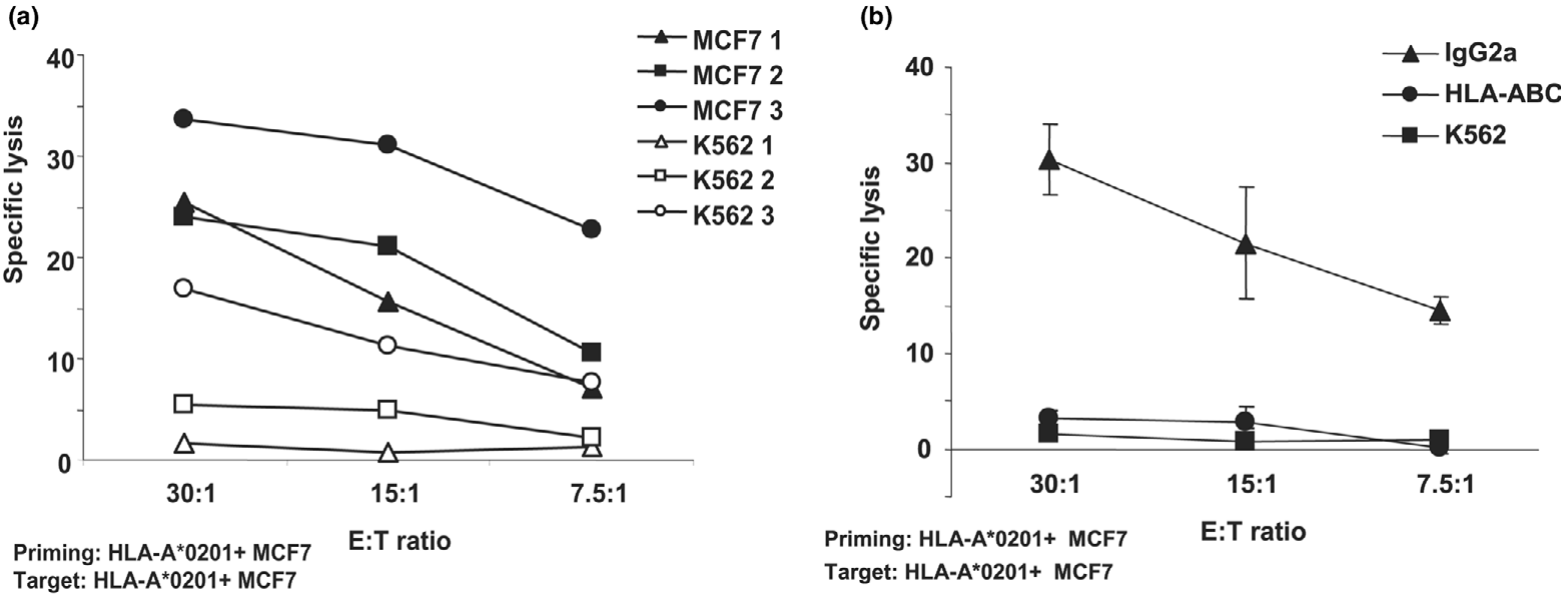
DCs loaded with killed breast cancer cells induce differentiation of effector CTLs. CD8^+^T cells primed, as described in Figure 1, were assessed in a standard ^51^Cr release assay using as targets the HLA-A*0201^+ ^MCF7 cells used to load DCs and NK sensitive K562 cells. **(a) **CD8^+ ^T cells primed by DCs loaded with MCF7 cell bodies exhibit cytotoxic activity against MCF7 used for priming but not against NK sensitive K562 cells. Three separate experiments are represented, indicated by different symbols: filled symbols indicate lysis of MCF7 breast cancer cells, and empty symbols indicate lysis of control K562 cells in the same experiment. **(b) **Cytotoxic activity was blocked by adding anti-MHC class I antibody (HLA-ABC) but not by adding relevant isotype control (IgG2a). Each value represents the mean from triplicate wells. Representative of three experiments performed. CTL, cytotoxic T lymphocyte; DC, dendritic cell; E:T, effector:target.

The cytotoxic effector function of antigen-primed CD8^+ ^T cells against breast cancer cells was analyzed in a 4-hour ^51^Cr release assay. As shown in Figure 2a, CD8^+ ^T cells cultured with HLA-A*0201^+ ^DCs loaded with killed HLA-A*0201^+ ^MCF7 breast cancer cells were able to kill MCF7 cells but not natural killer sensitive K562 cells. Such effective priming of tumor specific CD8^+ ^T was achieved with naïve T cells from three different donors. Tumor cell lysis could be abolished by pretreatment of the target MCF7 cells with anti-MHC class I antibody, thus confirming that induction of HLA class I restricted CTLs was taking place in these cultures (Figure 2b).

**Figure 3:**
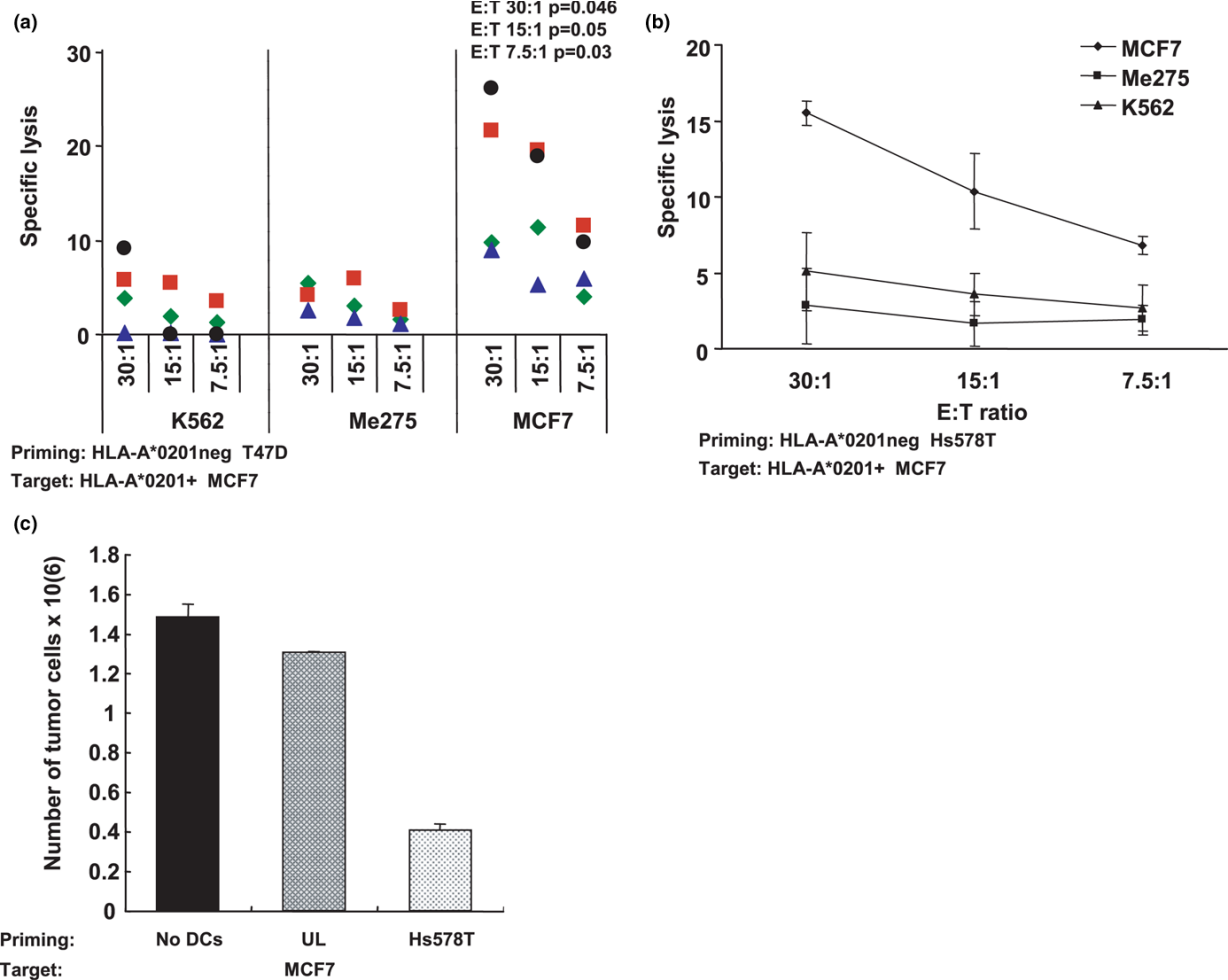
Cross-priming of breast cancer specific CTLs. HLA-A*0201^+ ^CTLs were generated as described in Figure 1, except that DCs were loaded with (panel a) HLA-A*0201^- ^T47D or (panels b and c) Hs578T breast cancer cells, and CTL activity was tested against HLA-A*0201^+ ^MCF7 breast cancer cells, HLA-A*0201^+ ^Me275 melanoma cells and NK cell sensitive K562 cells. **(a) **DCs loaded with killed HLA-A*0201^- ^T47D breast cancer cells induced CTLs that were able to lyse HLA-A*0201^+ ^MCF7 breast cancer cells but not HLA-A*0201^+ ^Me275 melanoma cells or NK cell sensitive K562 cells. The y-axis shows the specific lysis, and the x-axis shows the different E:T ratios. Data from four independent experiments are indicated by color code. A nonparametric Kruskal-Wallis comparison between the killing of three targets at different E:T ratios was conducted. **(b) **A similar experiment as is summarized in panel a, except CD8^+ ^T cells were primed with DCs loaded with killed Hs578T cells; shown are representatives of three experiments. Values are expressed as average and standard deviation of triplicate wells. **(c) **CD8^+ ^T cells cultured either with cytokines only (no DCs), with unloaded DCs (UL), or with DCs loaded with killed Hs578T breast cancer cells (Hs578T) were co-cultured for 24 hours with HLA-A*0201^+ ^MCF7 breast cancer cells in triplicates. Survival of MCF7 cells (x-axis) was calculated by Trypan blue exclusion. In each case, shown are the results of one representative experiment from two performed. CTL, cytotoxic T lymphocyte; DC, dendritic cell; E:T, effector:target; NK, natural killer.

To confirm that cross-priming had occurred, naïve HLA-A*0201^+ ^CD8^+ ^T cells were exposed to three stimulations with autologous HLA-A*0201^+ ^DCs loaded with HLA-A*0201^- ^breast cancer cell lines T47D or Hs578T and tested on HLA-A*0201^+ ^MCF-7 target cells in a 4-hour ^51^Cr release assay (Figure 3a and 3b) and a 24-hour tumor inhibition assay [24] (Figure 3c). As shown in Figure 3, CTLs primed in these cultures could kill HLA-A*0201^+ ^MCF7 breast cancer cells not used in the priming, thus demonstrating cross-priming against antigens shared between the priming and the target breast cancer cell lines (Figure 3a and 3b). These antigens were mostly breast cancer specific, as further demonstrated by the inability of the breast cancer primed CTLs to kill HLA-A*0201^+ ^Me275 melanoma cells (Figure 3a and 3b). These results were obtained with CD8^+ ^T cells isolated from four different healthy donors in four independent experiments. These data clearly show that DCs loaded with killed allogeneic breast cancer cells can cross-prime naïve CD8^+ ^T cells to differentiate into CTLs specific for antigens shared between breast cancer cell lines.

### Specificity of breast cancer cross-primed cytotoxic T lymphocytes for defined shared tumor antigens

The desired objective of loading DCs with killed breast cancer cells was to prime a polyclonal population of antigen specific CTLs. To determine whether this was indeed the case, we tested the primed CTL cultures for the presence of T cells specific for three well known tumor antigens expressed in breast cancers, namely cyclin B1, MUC-1 and survivin.

#### Cyclin B1-specific cytotoxic T lymphocytes

**Figure 4:**
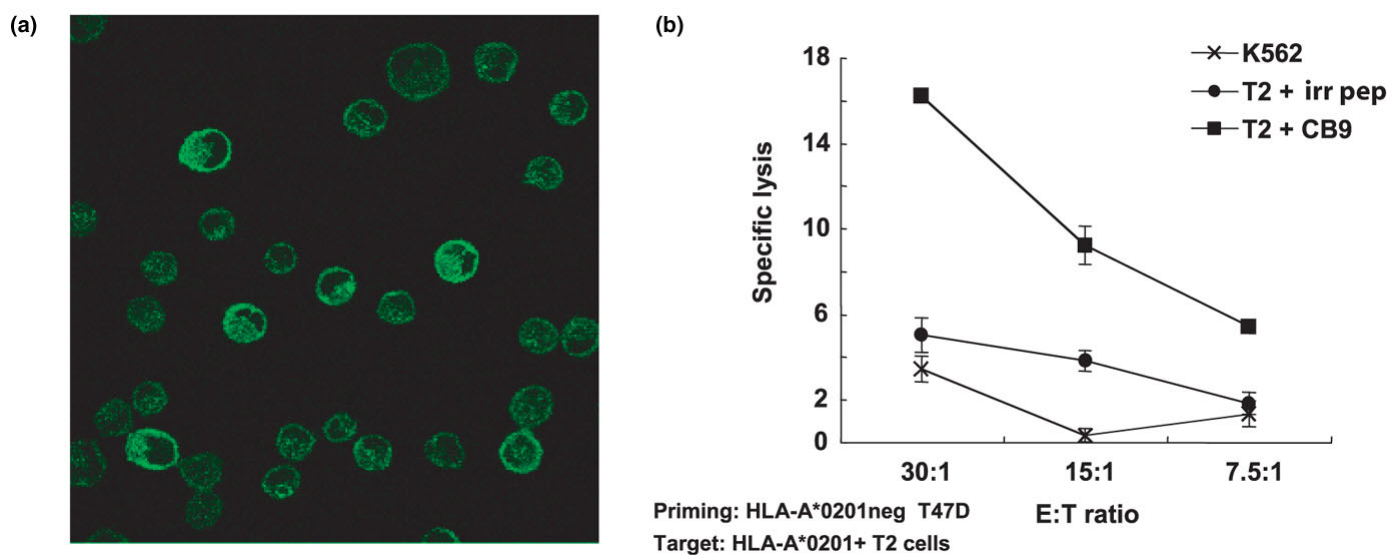
Cross-priming of cyclin B1 specific CTLs.**(a) **Fluorescence microscopy analysis of cyclin B1 staining with T47D breast cancer cells. No staining with isotype control could be seen. **(b) **CTLs primed as described in Figure 3 killed T2 cells pulsed with cyclin B1 derived CB9 peptide, but not T2 cells pulsed with irrelevant (PSA) peptide, indicating the presence of cyclin B1-specific CTLs. Shown are representatives of three experiments. Values are expressed as average and standard deviation of triplicate wells. CTL, cytotoxic T lymphocyte; DC, dendritic cell.

The discovery of cyclin B1 as a potential target for T cells in patients with breast as well as head and neck cancers expanded the spectrum of defined tumor antigens expressed in breast cancer [7]. Among the breast cancer cells we used, T47D cells demonstrated the greatest over-expression and cytoplasmic accumulation of cyclin B1 (Figure 4a). Thus, CD8+ T cells that were primed with DCs loaded with killed T47D cells were assayed for their capacity to kill cyclin B1 peptide pulsed T2 target cells. As shown in Figure 4b, T2 cells pulsed with the cyclin B1 peptide were killed efficiently whereas the control targets, namely T2 cells pulsed with irrelevant peptide or natural killer cell sensitive K562 cells, were killed at a significantly lower (background) level. These results were obtained with naïve CD8+ T cells from three different healthy donors and demonstrate successful cross-priming of antigen specific CTLs by DCs loaded with killed tumor cells.

#### MUC-1 specific cytotoxic T lymphocytes

**Figure 5:**
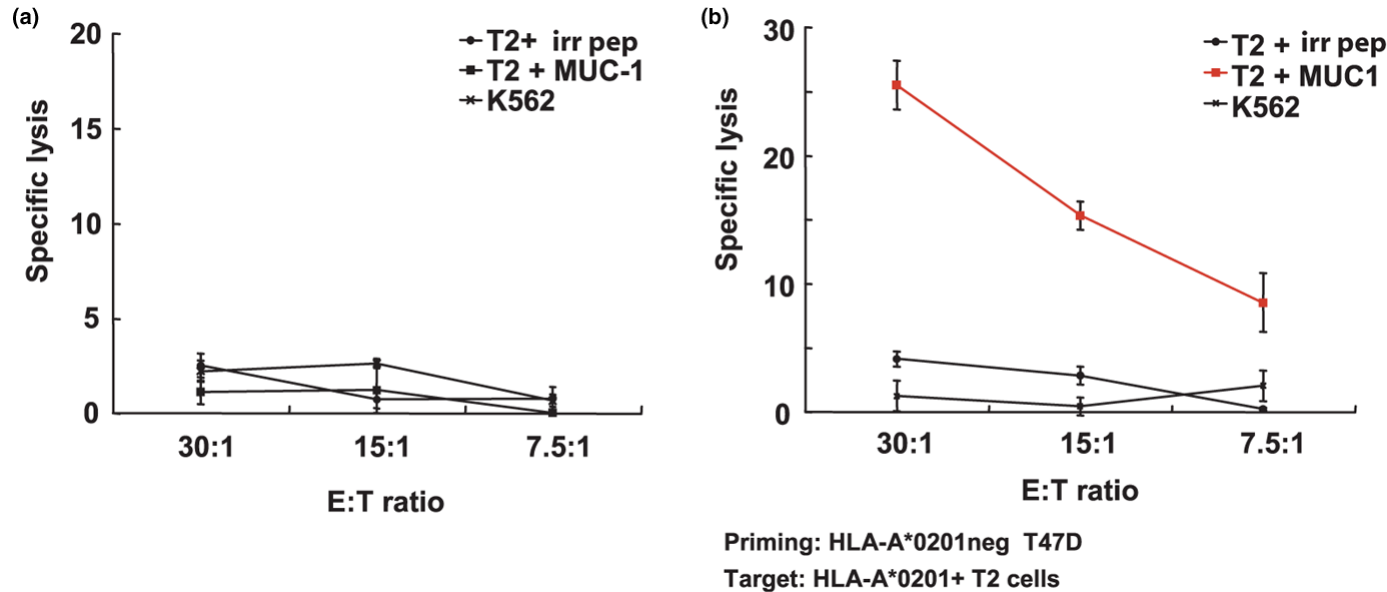
Cross-priming of MUC-1 specific CTLs. After the third stimulation of CD8^+ ^T cells by DCs loaded with killed T47D, CD8^+ ^T cells were stimulated with DCs loaded with MUC-1 peptide once and analyzed 5 days after last stimulation. The results of ^51^Cr release assay show the presence of MUC-1 specific CD8^+ ^T cells. The percentage cytotoxicity is measured as a function of spontaneous and total release. **(a) **Lack of killing of target cells expressing MUC-1 peptide after 3 weeks of culture with DCs loaded with killed breast cancer cells. **(b) **Killing of target cells after brief re-stimulation of the same cultures with DCs loaded with MUC-1 peptide, indicating the presence of primed T cells. Results are representative of five experiments, and each value represents the mean from triplicate wells. CTL, cytotoxic T lymphocyte.

T47D cells are also positive for the tumor antigen MUC-1, so we anticipated that the above cultures primed with T47D-loaded DCs would also yield T cells specific for MUC-1. This turned out not to be the case Figure 5a shows that MUC-1 peptide pulsed T2 cells were not killed. Earlier studies by some of us [25-27] demonstrated that MUC-1 glycoprotein, which is over-expressed and secreted by breast cancer cells, is endocytosed by DCs but is mostly retained in early endosomes; this leads to its inefficient processing and presentation to T cells, and hence a lower frequency of MUC-1 specific effector cells. We assumed that this was responsible for the low (undetectable) frequency of cross-primed effector CTLs, and in order to increase further the MUC-1 specific CTL frequency we gave the cultures one additional restimulation with DCs pulsed with a defined MUC-1 derived peptide. Thus, naïve CD8+ T cells were primed for 3 weeks with DCs loaded with killed T47D breast cancer cells, and at day 7, after the third stimulation, the T cells were restimulated for 5 days with MUC-1 peptide pulsed autologous DCs. As shown in Figure 5b, T cells given one additional restimulation with peptide-pulsed DCs were capable of significant lysis of MUC-1 peptide pulsed but not control T2 cells pulsed with irrelevant peptide. Control CD8+ T cells from 3-week cultures primed with control (not loaded) DCs and restimulated with peptide pulsed DCs were unable to expand MUC-1 specific T cells in this assay (<1% specific lysis at an effector:target ratio of 30:1). Thus, DCs loaded with killed allogeneic breast cancer cells can also prime (albeit at lower frequency) naïve CD8+ T cells to differentiate into MUC-1 peptide specific CTLs.

#### Survivin-specific T cells

**Figure 6:**
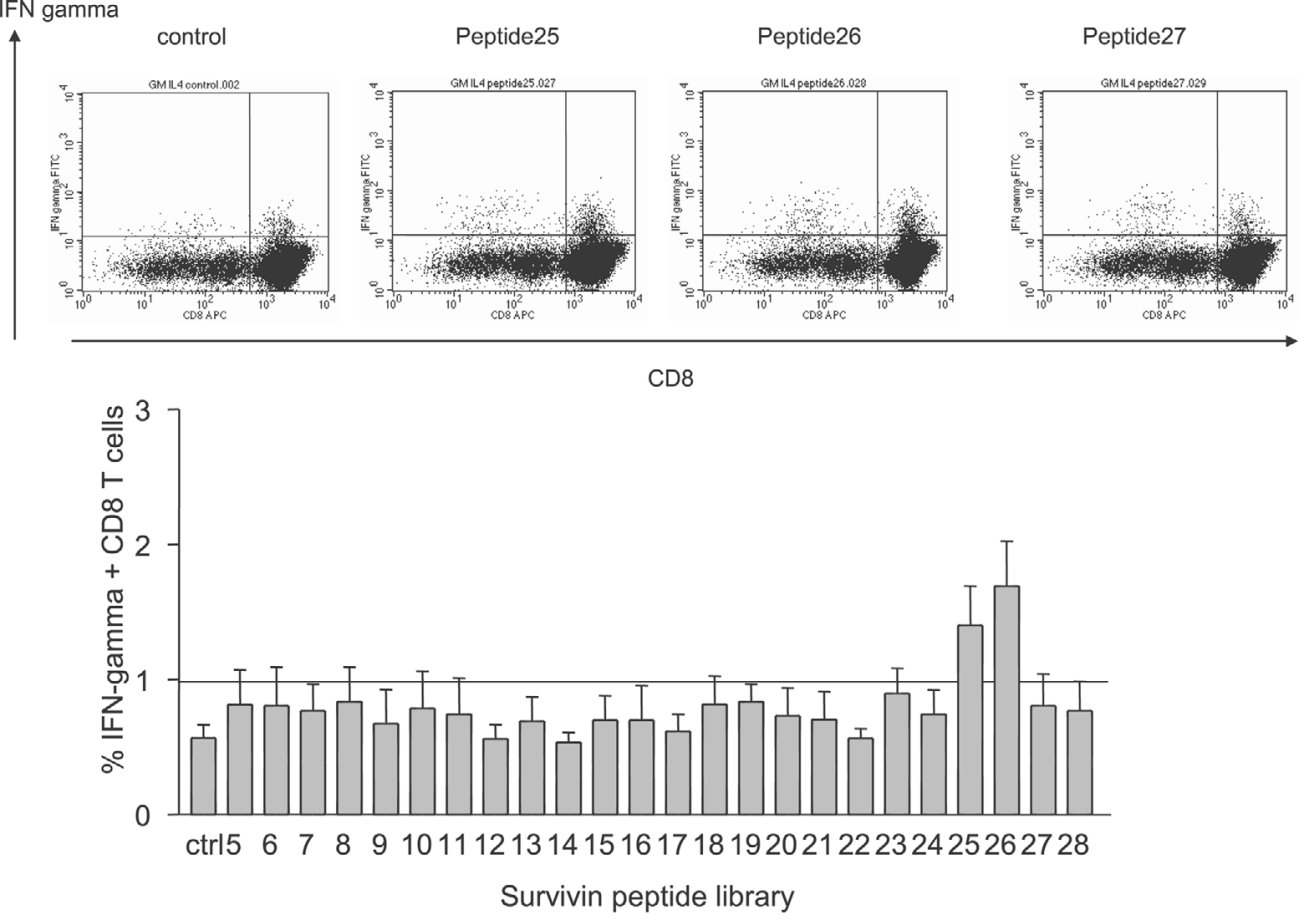
Cross-priming of survivin peptide-specific CTLs. HLA-A*0201^+ ^naïve CD8^+ ^T cells (CD45RA^+^CD45RO^-^CCR7^+^) were stimulated twice with autologous DCs loaded with killed allogeneic HLA-A*0201^+ ^MCF-7 breast cancer cells in 2-week cultures supplemented with IL-7 and IL-2 (10 U/ml). Seven days after the second stimulation, the T cells were harvested, washed, and restimulated once with autologous DCs loaded with an overlapping peptide library derived from survivin protein. After an additional 7 days of culture, T cells were cultured for 6 hours with DCs pulsed with survivin derived peptides (x-axis), Golgi stop was added in the last 4 hours of cocultures, and the presence of specific T cells was measured by intracytoplasmic staining with anti-IFN-γ mAb and flow cytometry. Percentage of IFN-γ producing CD8^+ ^T cells is shows on the y-axis; values are expressed as average and standard deviation from three independent experiments. CTL, cytotoxic T lymphocyte; DC, dendritic cell; IFN, interferon; IL, interleukin; mAb, monoclonal antibody.

To evaluate further the repertoire breadth of elicited CD8^+ ^T cell immunity, we assessed the priming of CD8^+ ^T cells against the shared tumor antigen survivin [28-30]. Here, we used another approach to analyze specificity and effector function (overlapping peptide libraries and intracytoplasmic IFN-γ staining), with the intention being to demonstrate priming to many different peptides derived from the same tumor antigen. Survivin positive HLA-A*0201^+ ^MCF7 killed breast cancer cells were used to load DCs. The T cells were assessed using a recall memory strategy as was used for the MUC-1 specific responses described above. Our expectation was that T cells specific for each individual peptide might be present in low frequency. Thus, after priming and boosting with DCs loaded with killed MCF7 breast cancer cells, the third T cell stimulation was carried out with DCs loaded with the library of overlapping 15-mer peptides representing the full length of survivin. Thereafter, the T cells were briefly (6 hours) restimulated with DCs loaded with survivin derived 15-mer peptides, stained with anti-IFN-γ mAbs and analyzed by flow cytometry (Figure 6). As shown in Figure 6, IFN-γ secretion was detected in T cells exposed to peptides 25 and 26. IFN-γ secreting T cells are predominantly those that label with anti-CD8 mAbs. Further analysis demonstrated that peptide 26 contained a 10 amino acid sequence consistent with the previously reported HLA-A*0201 restricted survivin peptide ELTLGEFLKL [29], and that peptide 25 contained an eight amino acid sequence (ELTLGEFL) from this peptide.

## Discussion

Using different target antigens and different assays to measure CTL function, we demonstrated that DCs loaded with killed allogeneic breast cancer cells cross-prime naïve CD8^+ ^T cells to differentiate into breast cancer specific effector CTLs. The elicited CTLs demonstrate specific effector function, as measured by their capacity to kill breast cancer cells used for priming, to kill T2 cells pulsed with defined peptides derived from breast cancer antigens, and to secrete IFN-γ upon peptide exposure. Among the primed CTLs, we demonstrate at least three specificities against the known shared breast tumor antigens cyclin B1, MUC-1, and survivin.

Interestingly, we observed differences in the priming of CD8^+ ^T cells with distinct specificities that were consistent with the unique nature of each antigen. Thus, T cells specific for cyclin B1 could be detected after priming and two rounds of boosting with DCs loaded with killed breast cancer cells. However, this strategy did not permit detection of MUC-1 specific T cells, which required an additional boost with MUC-1 peptide pulsed DCs. We ascribe this to the differences in efficiency of presentation of different antigens by loaded DCs and/or differences in the frequency of precursor T cells with these specificities. Indeed, cyclin B1 represents a protein antigen whose function as a tumor antigen is associated with increased cytoplasmic accumulation followed by degradation through ubiquitination [7]. This might make it more accessible to the DC antigen processing/presentation machinery than the transmembrane MUC-1 glycoprotein. Previous studies by some of us [25-27] demonstrated that MUC-1 and HER-2/neu are retained in DC early endosomes, leading to inefficient processing and cross-presentation to T cells. These observations, together with our earlier study and the lack of specificity to some other defined breast cancer antigens, including HER-2/neu [23], suggest that efficient cross-presentation of individual tumor antigens by DCs loaded with killed breast cancer cells will depend on the nature of these antigens or the form in which they are taken up by DCs. For example, although we found that DCs loaded with whole tumor cells are not efficient in cross-presenting MUC-1, a recent study [31] demonstrated that loading DCs with breast cancer cell lysates permits more efficient priming of MUC-1 specific CD8^+ ^T cells. It is difficult to determine whether the difference in the outcomes between that study and ours is due to a real difference between loading DCs with whole tumor cells and loading them with tumor cell lysate, or to a difference in how the MUC-1 specific T cells were detected. CD8^+ ^T cells in that report were measured based on tetramer binding but in our study measurement was based on a functional assay, specifically the activity of CTLs against target cells expressing MUC-1 peptide.

Thus, our data offer experimental proof that using DCs loaded with killed allogeneic breast cancer cells for antigen presentation leads to priming of T cells to multiple tumor antigens, some that are already known and could be tested here, and potentially many more unknown but nevertheless important shared tumor antigens. The advantage of this approach is the ability to generate, with one vaccine, T cells specific for many tumor antigens and in many patients, regardless of HLA type. Although it is conceivable that part of the generated response might be due to HLA mismatch, breast cancer tumor antigen specific immune responses were also generated. Finally, we previously demonstrated the validity of this approach in patients with stage IV melanoma [32]. In that study patients were vaccinated with DCs loaded with killed allogeneic melanoma cells. We that melanoma specific immune responses were induced and, perhaps more importantly, durable (>33 months) objective clinical responses were identified in 10% of patients in whom conventional therapy failed. Thus, this vaccination strategy might now be tested in phase I studies in patients with metastatic breast cancer.

## Conclusion

This ability of DCs loaded with killed allogeneic breast cancer cells to elicit multiantigen specific immunity supports their use as vaccines in patients with breast cancer.

## Abbreviations

CTL = cytotoxic T lymphocyte; DC = dendritic cells; IFN = interferon; IL = interleukin; mAb = monoclonal antibody.

## Competing interests

Dr. Jacques Banchereau has been a consultant for Argos Therapeutics and is a scientific founder of ODC Therapy, Inc., two private companies, and has stock-options for both. Dr. Anna Karolina Palucka is a scientific founder of ODC Therapy, Inc., a private company, and has stock-options. These private companies are related to dendritic cell vaccines. Neither one of them has in any way supported the study, the results of which are described in the current manuscript.

## Authors' contributions

HS designed and performed the experiments, analyzed the data, and wrote the manuscript. PD designed and performed experiments, and analyzed the data. CD performed experiments and analyzed the data. OJF designed experiments, analyzed the data, and wrote the manuscript. JB designed experiments, analyzed the data, and wrote the manuscript. AKP led the study, designed the experiments, analyzed the data, and wrote the manuscript.
